# Expression and Distribution of Mesencephalic Astrocyte-Derived Neurotrophic Factor in the Retina and Optic Nerve

**DOI:** 10.3389/fnhum.2016.00686

**Published:** 2017-01-19

**Authors:** Feng-Juan Gao, Sheng-Hai Zhang, Ting-Ting Li, Ji-Hong Wu, Qiang Wu

**Affiliations:** ^1^Department of Ophthalmology, Shanghai Jiao Tong University Affiliated Sixth People's HospitalShanghai, China; ^2^Eye & ENT Hospital, State Key Laboratory of Medical Neurobiology, Institutes of Brain Science and Collaborative Innovation Center for Brain Science, Shanghai Medical College, Fudan UniversityShanghai, China; ^3^Shanghai Key Laboratory of Visual Impairment and RestorationShanghai, China; ^4^Key Laboratory of Myopia, Ministry of Health, Fudan UniversityShanghai, China

**Keywords:** mesencephalic astrocyte-derived neurotrophic factor, retinal ganglion cells, retina, optic nerve, distribution

## Abstract

Mesencephalic astrocyte-derived neurotrophic factor (MANF), otherwise named Arginine-Rich, Mutated in Early-stage Tumors (ARMET), is a secretory endoplasmic reticulum stress (ERS) protein that is widely expressed in mammalian tissues. To date, little is known about the distribution and expression of MANF in the retina and optic nerve (ON). Therefore, we studied the expression and distribution of MANF in the ON and retina by real-time PCR, immunofluorescence staining and western blotting. Results from rat and mouse were highly consistent in the retina. MANF was detected in both tissues in rat, wherein it was principally localized to the ganglion cell layer (GCL), followed by the inner nuclear layer (INL). The MANF protein levels in the rat retina were 3.33-fold higher than in the rat ON. Additionally, MANF was robustly expressed by retinal ganglion cells (RGCs) in the human retina. In human ON, MANF was partially co-localized with glial fibrillary acidic protein (GFAP), suggesting that it was not restricted to astrocytes. *In vitro* studies confirmed that MANF could be robustly expressed in RGCs and was found principally within the cytoplasm. Hypoxia can stimulate up-regulation by of MANF expression over time, suggesting that MANF may play a vital role in the functional regulation of RGCs both in health and disease. We believe that the present study improves our understanding of the distribution and expression of MANF in the retina and ON and could help in further analysis of its interact and correlate with the relevant ophthalmic diseases.

## Introduction

Mesencephalic astrocyte-derived neurotrophic factor (MANF), also known as Arginine-Rich, Mutated in Early-stage Tumors (ARMET), is a secreted endoplasmic reticulum stress (ERS) protein (Apostolou et al., 2008). It was first isolated from a rat mesencephalic type-1 astrocyte cell line. MANF together with its homologous protein, cerebral dopamine neurotrophic factor (CDNF), forms a novel family of conserved secreted neurotrophic factors that are structurally and functionally different from classical neurotrophic factors' families (Lindholm and Saarma, 2010; Glembotski et al., 2012). Structural analysis indicated that MANF possesses a secretion signal without a pro-sequence, indicating that it can be activated without being enzymatically processed.

MANF can be induced by ischemic, hypoxic, or epileptic damages in rodent secretory cells and tissues, including pancreatic β cells, brain and heart tissue (Lindholm et al., 2008; Tadimalla et al., 2008). Addition of recombinant MANF or overexpression of MANF can not only increase cell viability and affect cell size and morphology but can also inhibit ERS-induced cell death and cell proliferation in non-neuronal cells (Apostolou et al., 2008). Moreover, MANF can be selectively neuroprotective roles to dopaminergic neurons vs. GABAergic or serotonergic neurons both *in vitro* and *in vivo* (Petrova et al., 2003; Lindholm et al., 2007), suggesting that it could be used for the remedy of neurodegenerative disorders. Recent studies found that MANF is also involved in the regulation of inflammatory response (Zhao et al., 2013; Chen et al., 2015). However, whether it also plays an important role in ocular tissue is yet unknown.

To study its function in the eye, it is important to first clarify its expression and distribution. In rodents, MANF and CDNF are expressed widely in various types of neuronal and non-neuronal tissues (Lindholm et al., 2007, 2008)— common characteristic for all neurotrophic factors (Sariola, 2001). Besides, MANF expression has already been studied in zebrafish, where it is expressed widely in the nervous system as well as in adult organs during both development and in adulthood (Chen et al., 2012). The widespread expression of MANF together with its regulation by cellular insults and evolutionary conserved nature implies that it likely has important roles in many tissue types, including the eyes, and could hence present a potentially novel therapeutic modality for the treatment of hypoxic-ischemic or neurodegenerative diseases of the retina. Thus far, however, to our best knowledge, there are no reports regarding the distribution and expression of MANF in the eye. Therefore, we applied a combination of real-time PCR, immunofluorescence staining and western blotting to determine the expression and distribution of MANF in the retina and optic nerve (ON).

## Materials and methods

### Ethics statement

All animal experiments conformed to the ARVO Statement for the Use of Animals in Ophthalmic and Vision Research. Human ocular tissue were obtained from recently deceased persons, with no known systemic disease, form the Red Cross Eye Bank, Eye and ENT Hospital of Fudan University following the guidelines of the Shanghai Clinical Human Research Ethics Committee. All processes adhered to principles of the Declaration of Helsinki, and the experiment was approved by the Office of Research Ethical Committee, Fudan University, and all endeavors were done to minimize animals' suffering.

### Source and processing of rodent and human tissues

Adult Sprague–Dawley rats (*n* = 10, weight, ~250 g) and C57BL/6 mice (*n* = 10, 4 months old) were raised under constant 12-h light/dark cycles and supplied with a standard rodent food and water *ad libitum*. An overall number of 10 rats and 10 mice were used in the study. Three human ocular tissues were used in this study, they were from Chinese donors of 38, 17, and 41 years old who had been screened to ensure there was no underlying ocular disease.

All animals were killed by an overdose of 10% chloral hydrate. The retinas and ONs were separated immediately and used for western blotting. Globes and ONs that were subsequently used for immunofluorescence staining were immersion-fixed in 4% paraformaldehyde overnight at 4°C. Then, the eyes were sequentially placed in 20 and 30% sucrose.

### Purified retinal ganglion cell (RGC) isolation, culture, and treatment

We followed our previously described two-step immunopanning method to isolate purified RGCs (Gao et al., 2016). Briefly, 2-day-old Sprague-Dawley rats were killed to obtain the retinas. The retinas were removed and dissociated in 4.5 U/mL of papain solution (Worthington, Lakewood, NJ). The cell suspensions were then transferred to a petri dish coated with rabbit anti-macrophage antibody (Cedarlane Laboratories, Ontario, Canada) and mouse anti-Thy1.1 antibody (Abcam, Cambridge, MA) successively. The adherent cells were collected and then seeded into 24 and 6-well plates pretreated with poly-D lysine (Sigma-Aldrich, St. Louis, MO) and mouse-laminin (Trevigen Inc., Gaithersburg, MD). Plates were incubated in a humidified incubator with 5% CO_2_ at 37°C. Immunocytochemical staining of Thy1.1 was performed after the neonatal RGCs were cultured for 3–4 days to determine the RGCs purity. Forty-eight hours after seeding, the RGCs were incubated with 200 μM cobalt chloride (CoCl_2_) for 24 or 48 h (Balaiya et al., 2012; Du et al., 2013; Kim et al., 2013; Supplementary Figure 1).

### Nuclear-cytoplasm protein separation and western blot analysis

Cytoplasm and nuclear proteins was separated using a nucleus-cytoplasm seperation kit (Nanjing Jiancheng Bioengineering Institute, Zhejiang, China) according to the manufacturer's instructions. Tissues and RGC cell proteins were extracted using a lysis buffer (Cell Signaling Technology, Danvers, MA, USA) supplemented with a protease inhibitor cocktail (Sigma-Aldrich, St. Louis, MO, USA). Protein concentrations were determined using the bicinchoninic acid assay (Sigma-Aldrich, St. Louis, MO, USA). Each protein preparation (15–20 μg) was separated on 12% denaturing sodium dodecyl sulfate-polyacrylamide gel electropheresis (SDS-PAGE), and the separated proteins were electrotransferred to polyvinylidene difluoride membranes (Millipore, Billerica, MA). After blocking with 5% non-fat milk for 1 h, the membranes were incubated overnight at 4°C with primary antibodies against rabbit anti-MANF (1:500 dilution, Abcam, Cambridge, MA), rabbit anti-Brn 3a (1:800 dilution, Abcam, Cambridge, MA), mouse anti-Thy1.1 (1:400 dilution, Abcam, Cambridge, MA), rabbit anti-GAPDH (1:1000 dilution, Abcam, Cambridge, MA) and mouse anti-β-actin (1:1000 dilution, Abcam, Cambridge, MA) (for internal control). Secondary antibodies included HRP-conjugated goat anti-rabbit antibody (Millipore, Massachusetts, USA) and HRP-conjugated goat anti-mouse antibody (Millipore, Massachusetts, USA). The blots were exposed to X-ray film (Hyperfilm ECL, Thermo Fisher Scientific, Rockford, IL, USA) and analyzed using the Kodak Imaging System (Kodak 440CF). The intensity of the band was quantified by densitometry using ImageJ software (NIH, Bethesda, MD, USA).

### Immunofluorescence

Immunofluorescence was detected on frozen 8 μm retinal sections and RGCs. Briefly, retinal and ON sections and the RGCs were incubated with rabbit anti-MANF (1:200, Abcam, Cambridge, MA) and mouse anti-glial fibrillary acidic protein (GFAP, 1:1000)/neuronal class III β-tubulin (TUJ1, 1:500)/ Thy1.1 (1:500, Abcam, Cambridge, MA) at 4°C overnight. Then, they were incubated with fluorescein-conjugated goat anti rabbit or mouse secondary antibody (1:400, Molecular Probes, USA) for 1 h at room temperature. In addition, slides were just incubated with fluorescein-conjugated goat anti rabbit or mouse IgG without primary antibodies to exclude non-specific binding. The slides were visualized and photographed by confocal microscopy (Leica SP8, Hamburg, Germany).

### Quantitative real-time PCR analysis

Total RNA was extracted using TRIzol reagent (Invitrogen, Carlsbad, CA, USA) and cDNA was generated using a SuperScript First-Strand Synthesis kit (Takara, Tokyo, Japan) according to the manufacturer's instructions. The gene-specific primers for β-actin and MANF were verified before use. Real-time PCR (7500 Fast, Applied Biosystems) was performed in duplicate with 10 ng of cDNA and 10 pmol of each primer. The primer sequences used were as follows: rat MANF (forward: 5′-CACTTTAGCGATTACAGGAAGG-3′, reverse: 5′- GGGACAGATTGAAGGCTGA-3′); rat β-actin (forward: 5′-CACCCGCGAGTACAACCTTC-3′, reverse: 5′-CCCATACCCACCATCACACC-3′). The PCR conditions were 10 min at 95°C followed by 40 cycles of 15 s at 95°C, 60 s at 60°C and 60 s at 72°C. The specificity of the detected signals was confirmed with a dissociation curve that consisted of a single peak. Using the SYBR green data, the relative RNA expression was normalized to β-actin. All samples were run in triplicate in each experiment. The data were analyzed using the 2^−ΔΔCT^ method.

### Statistical analysis

Each experiment was performed at least 3 times; all data are expressed as the mean ± standard deviation. The data were analyzed using STATA 7.0 software. The Student's *t*-test was used for analysis of mRNA and MANF protein expression between two groups. *P* < 0.05 was considered statistically significant.

## Results

Retinal and RGC samples incubated with secondary antibodies without primary antibody displayed little or no autofluorescence (Supplementary Figure 2).

### Expression of MANF in the rodent retina and ON

**Figure 1:**
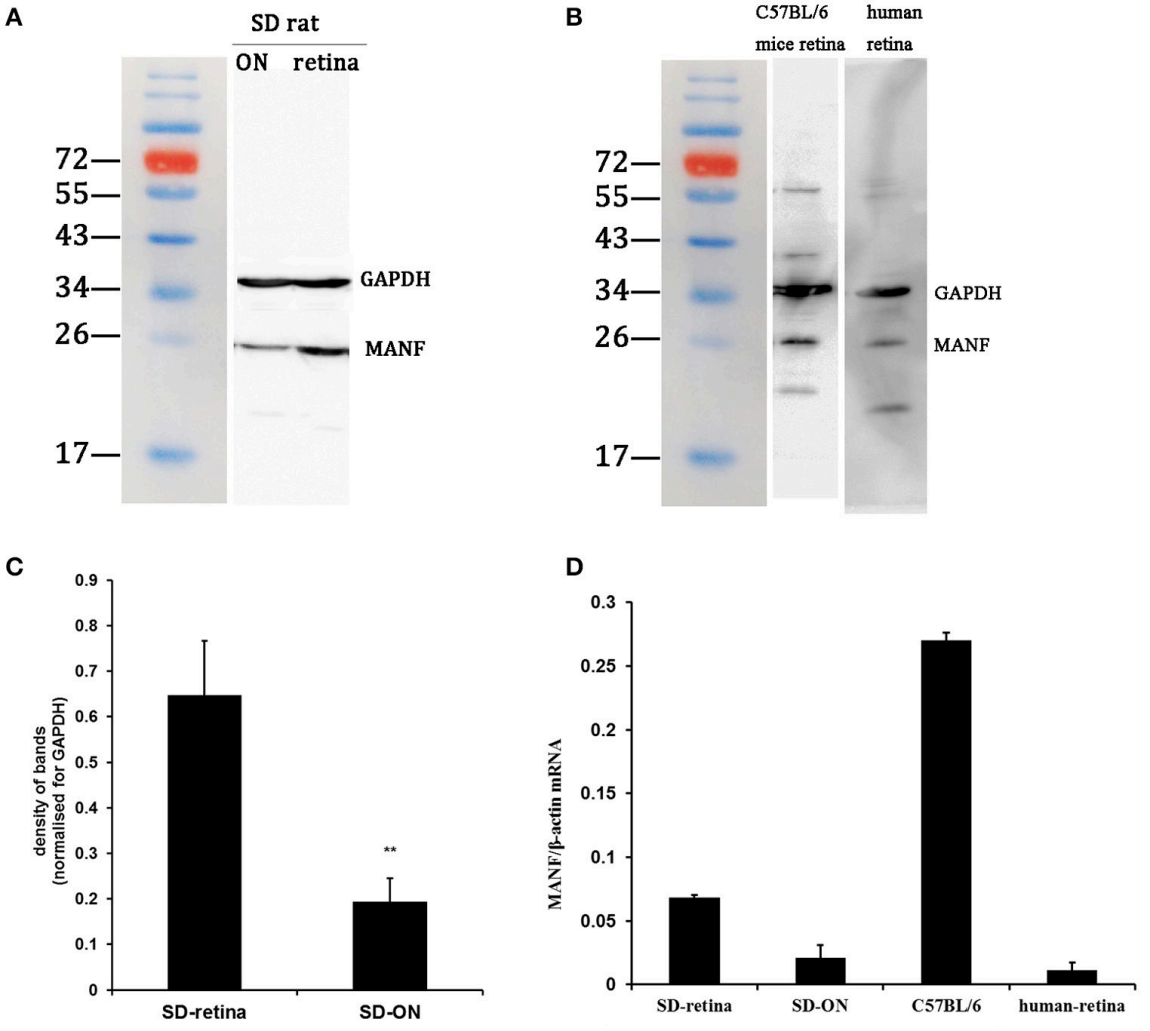
**Western blot and real-time PCR analysis of MANF expression in the retina and optic nerve (ON). (A)** In tissue extracts from SD rats, a major band of the expected molecular weight (25 kDa) is clearly seen. **(B)** In tissue extracts from C57BL/6 mice and human retinas, a major band of the expected molecular weight is seen. **(C)** Quantification of the MANF protein levels: the levels were higher in the retina vs. the ON when normalized for GAPDH (mean ± SEM, *n* = 6). ^**^P < 0.01. **(D)** The expression levels of the MANF mRNAs relative to the housekeeping gene β-actin in the SD rat, C57BL/6 mouse, and human retina and in the SD rat ON.

Western blotting was used to find out if MANF could be expressed in the rodent retina and ON. Figures 1A,B show that MANF was present in the both tested tissues of both rats and mice: a single major protein band of the expected 25-kDa molecular size was clearly seen on the membrane. Densitometry indicated that the expression of MANF in rat retina was significantly higher than in ON when standardized with GAPDH (*P* < 0.01). The protein levels of MANF were 3.33-fold higher in the rat retina than in the ON (Figure 1C). The expression levels of MANF mRNAs in the ON and retina relative to the housekeeping gene actin are shown in Figure 1D. Indeed, MANF mRNAs are expressed in relatively high amounts in both mouse and rat retinas. The presence of both MANF protein and mRNA suggest that MANF is expressed in the rodent retina and ON.

### Distribution of MANF in the rodent retina

**Figure 2:**
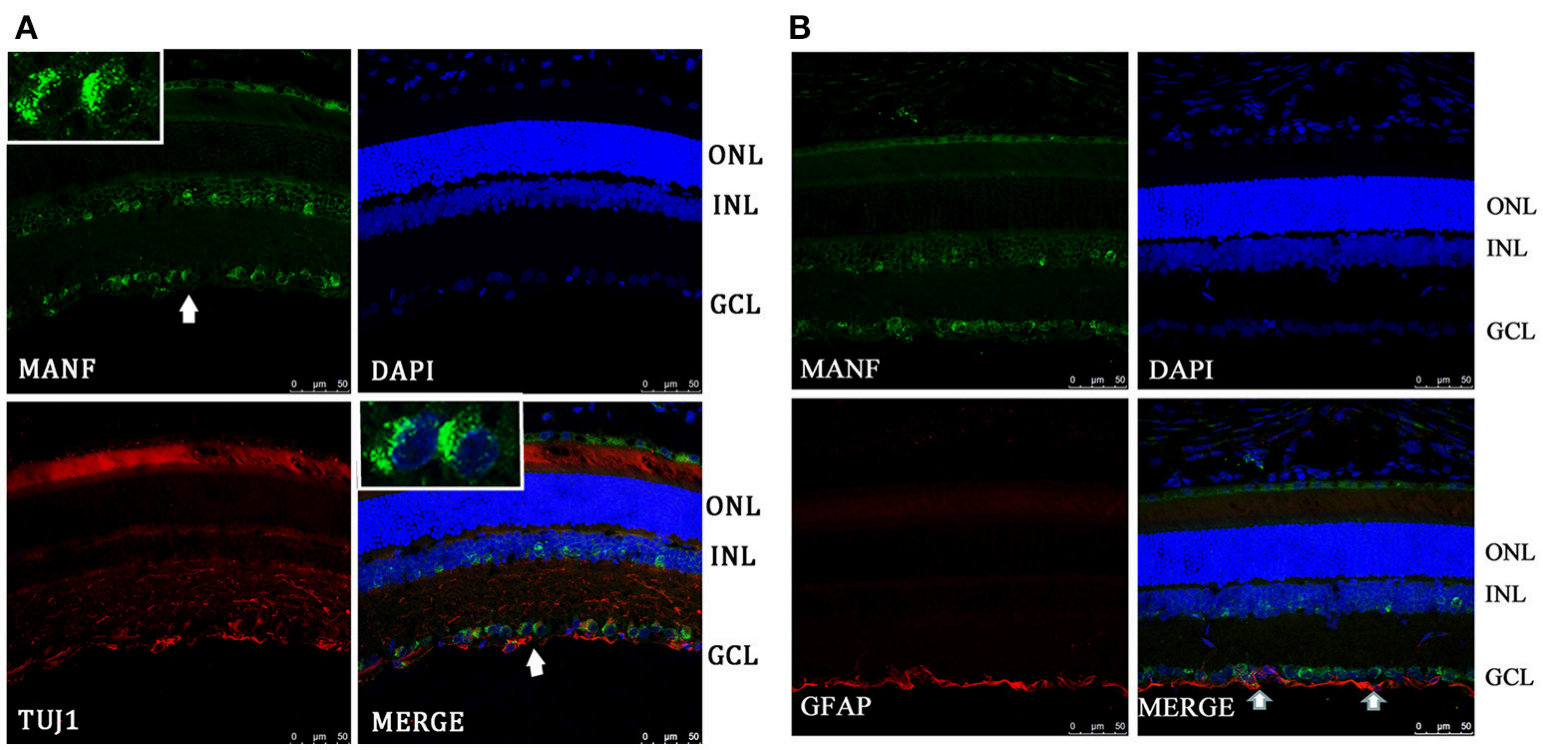
**Immunofluorescence of the MANF protein in SD rat retina (green for MANF; red for TUJ1 and GFAP). (A)** is double labeled for MANF and TUJ1. Images at the top left of the white box show high magnification of the cells indicated by white arrows. Fluorescence staining shows that MANF fluorescence intensity in the cytoplasm is much stronger than in the nucleus. **(B)** is double labeled for MANF and GFAP. MANF staining is intensely distributed in the cell membrane and cytoplasm in the GCL and with less intensity in the INL. Moderate MANF immunoreactivity is observed in the ONL. MANF is partially co-localized with the glial cell marker GFAP in the GCL (indicated by arrows). The nuclei are labeled with DAPI (blue). Scale bar: 50 μm. ONL: outer nuclear layer; INL: inner nuclear layer; GCL: ganglion cell layer.

**Figure 3:**
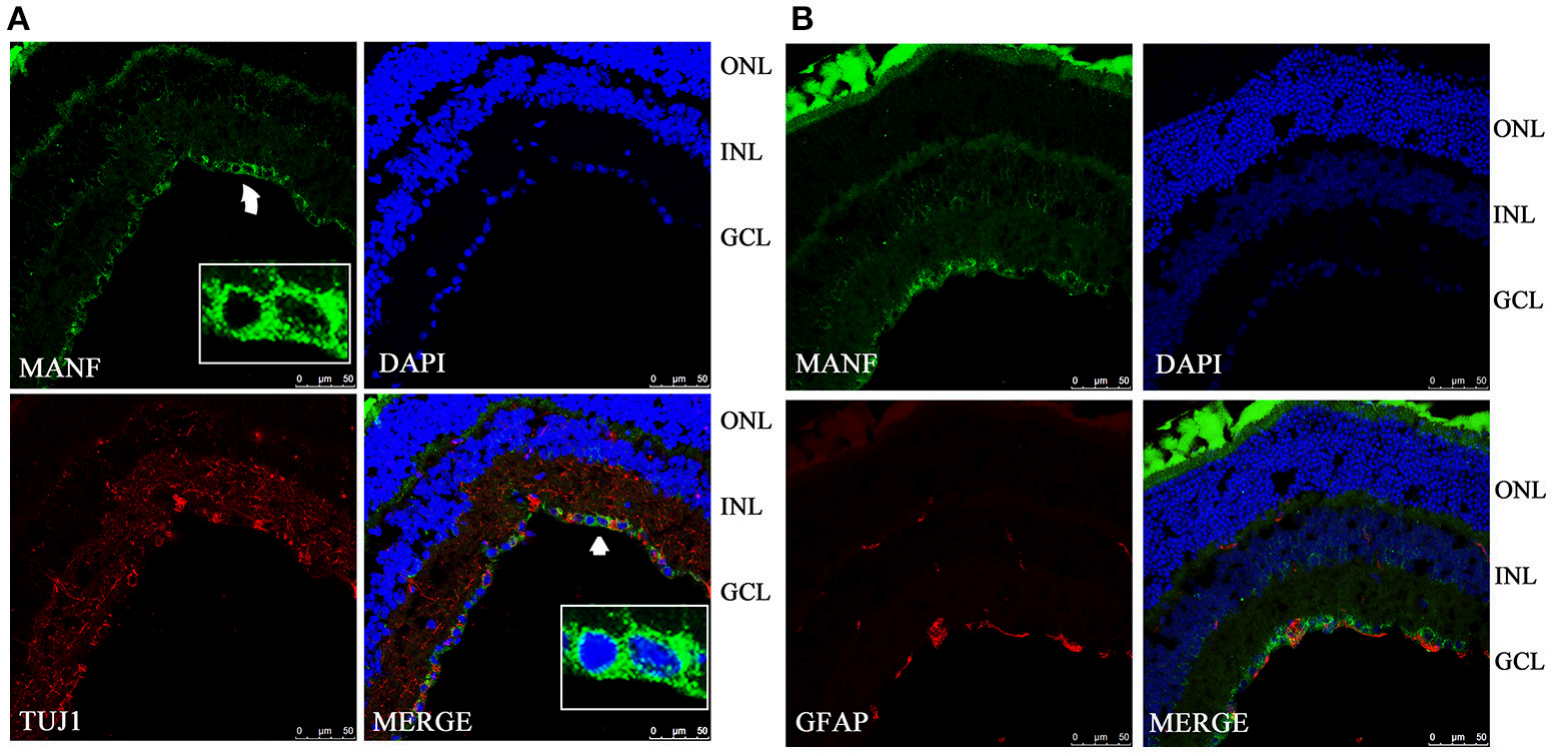
**Immunofluorescence of the MANF protein in the C57BL/6 mouse retina (green for MANF; red for TUJ1 and GFAP). (A)** is double labeled for MANF and TUJ1. Images at the bottom right of the white box show high magnification of the cells indicated by white arrows. Fluorescence staining shows that MANF fluorescence intensity in cytoplasm is much stronger than in the nucleus. **(B)** is double labeled for MANF and GFAP. MANF staining is intensely distributed in the cell membrane and cytoplasm in the GCL and with less intensity in the INL. Moderate MANF immunoreactivity is observed in the ONL. The nuclei are labeled with DAPI (blue). Scale bar: 50 μm. ONL: outer nuclear layer; INL: inner nuclear layer; GCL: ganglion cell layer.

To validate and extend the findings from the results of western blots, we determined the expression pattern of MANF in the rodent retina and ON by immunofluorescence staining. Similar results were obtained with the rat and mouse retina. MANF is predominantly localized to the ganglion cell layer (GCL), while inner nuclear layer (INL) showed only mild staining. As TUJ1 is usually considered as a protein marker of RGCs that stain neuronal axons (Morishita et al., 2014) and GFAP is present only in astrocytes in the GCL, double immunofluorescence staining was done for MANF with GFAP and TUJ1 for further cellular localization. The results showed that the MANF-positive band in the GCL was present in parts of both the two kind of cells (Figure 1), with greater number of MANF and TUJ1-positive cells in GCL confirmed through quantitative analysis (*P* < 0.01, Figures 2, 3, **5A**). This indicated that MANF was expressed robustly by RGCs but only limitedly by astrocytes. At higher magnification, it was seen that MANF was preferentially concentrated in the cytoplasm of the soma, with weak labeling in the nucleus (Figures 2, 3); however, no immunoreactivity was visible in the outer nuclear layer (ONL).

### Expression of MANF in human retina and ON

**Figure 4:**
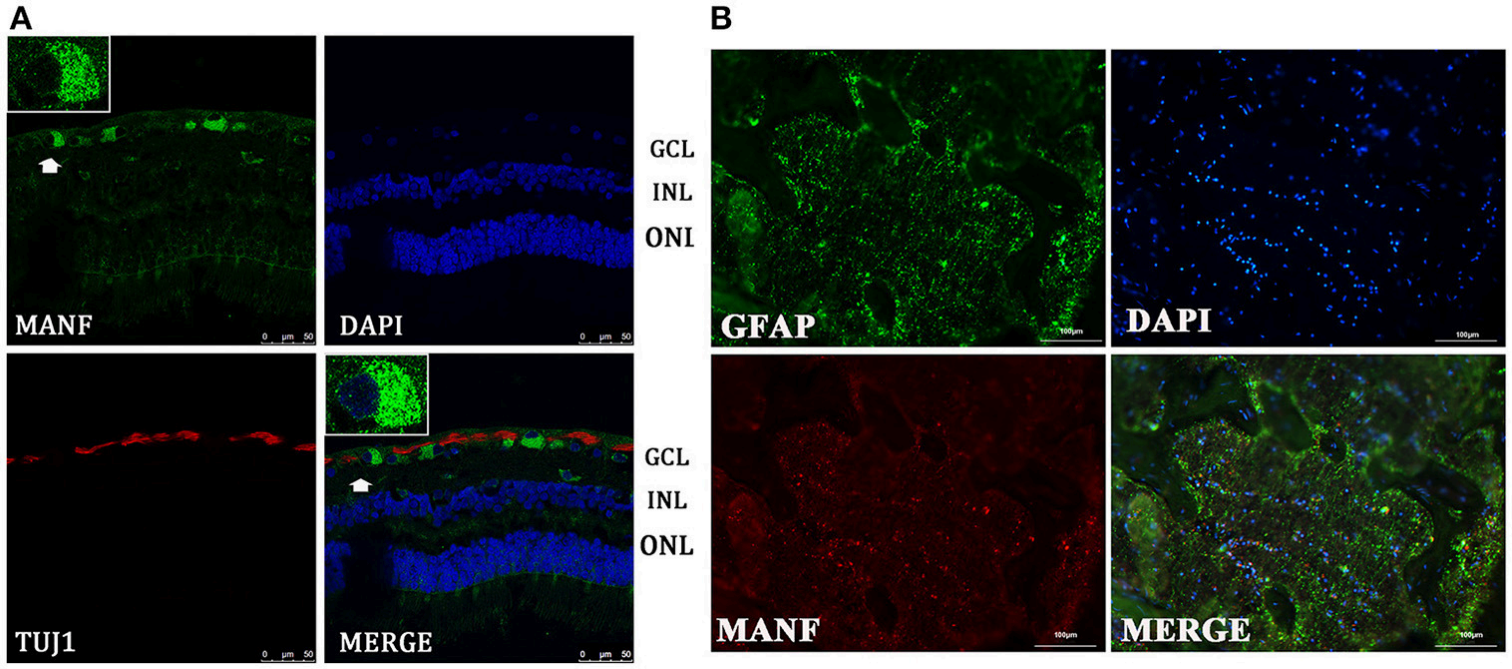
**Immunofluorescence of the MANF protein in the human retina and ON. (A)** is double labeled for MANF (green) and TUJ1 (red) in retina. MANF staining is intensely distributed in the cell membrane and cytoplasm in the GCL. Moderate MANF immunoreactivity is observed in the INL and ONL. Images at the top left of the white box show high magnification of the cells indicated by white arrows. Fluorescence staining shows that MANF fluorescence intensity in the cytoplasm is much stronger than in the nucleus. Scale bar: 50 μm. **(B)** is double labeled for MANF (red) and GFAP (green) in the ON. MANF is partially co-localized with GFAP. The nuclei are labeled with DAPI (blue). Scale bar: 100 μm. ONL: outer nuclear layer; INL: inner nuclear layer; GCL: ganglion cell layer.

Western blot analysis revealed that MANF was also present in human retinas, as the anti-MANF antibody detected a single major protein band of 25-kDa (Figure 1B). Quantitative real-time PCR analysis showed that relatively high amounts of MANF mRNA were expressed in the retina. MANF immunofluorescence staining of the optic nerve shows that MANF could be expressed in the ON and was partially co-localized with GFAP, suggesting that MANF could be expressed by other cell types except for astrocytes (Figure 4B).

### Distribution of MANF in the human retina

**Figure 5:**
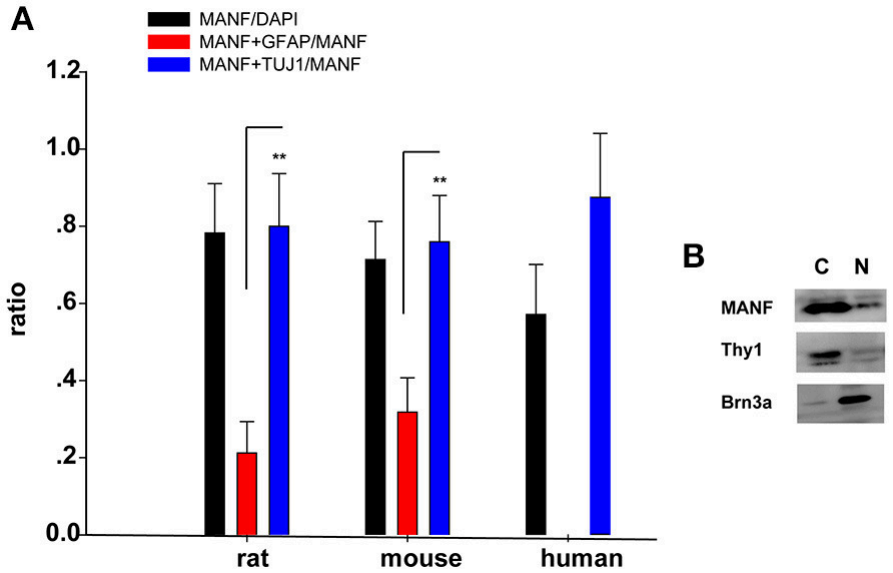
**(A)** Quantification of MANF and TUJ1/GFAP-positive cells. The Y-axis is the ratio of cells positive to MANF/DAPI, MANF+TUJ1/MANF, and MANF+GFAP/MANF. The quantitative values are expressed as mean ± standard deviation. ^**^P < 0.01, *t*-test; MANF+TUJ1/MANF group compared with the MANF+GFAP/MANF group. **(B)** Western blot analysis of MANF expression in nuclear and cytoplasmic fraction of RGCs. Antibodies against Brn3a and Thy1 were separately used in order to check nuclear and cytoplasmic fraction enrichment. N: nuclear; C: cytoplasm.

We performed retinal immunofluorescence staining, to better understand the distribution of MANF protein in the retina. Confocal analysis showed that MANF was widely distributed in the GCL, where it was preferentially distributed around the cytoplasm but poorly in nucleus (Figure 4A), suggesting that MANF is largely localized in the cytoplasm. Next, we quantified the number of MANF positive cells in the GCL, 87.5 ± 16.57% of cells showed positive stained both by MANF and TUJ1 immunofluorescence relative to MANF single positive, 57.14 ± 12.98% of cells with MANF relative to all cells in the RGC layer (Figure 5A). Moreover, only mild staining was observed in the cell bodies of the INL and ONL. Taken together, our data suggest that MANF is largely expressed in the cytoplasm of RGCs in the human retina.

### *In vitro* validation and changes of MANF expression in RGCs with hypoxia

**Figure 6:**
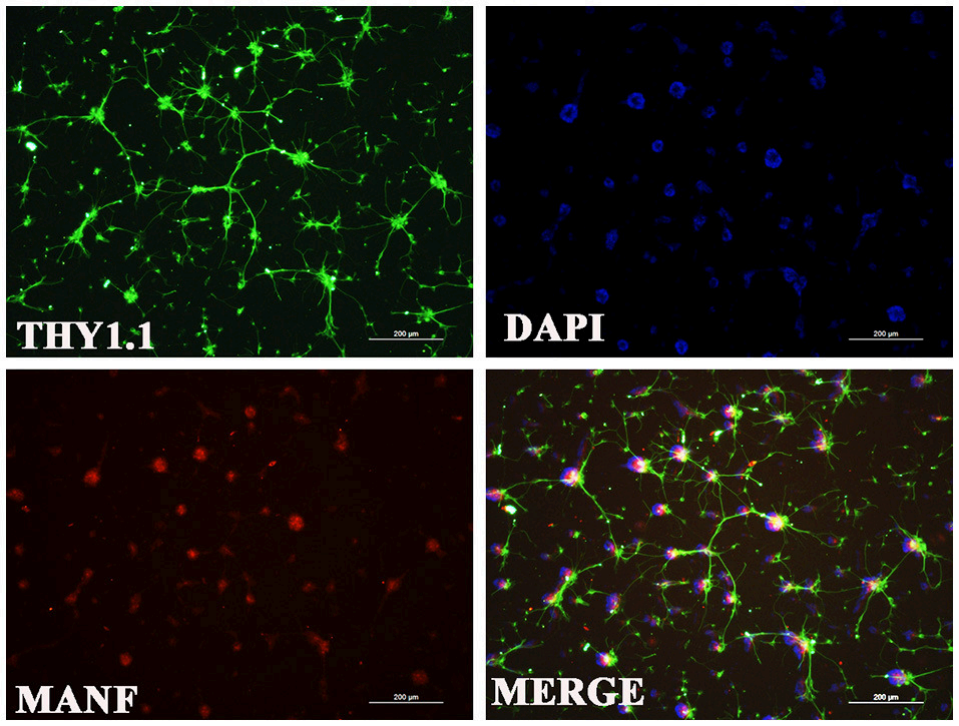
**Immunofluorescence of the MANF protein (red) in rat RGC cultures**. RGCs were identified with Thy1.1 (green). The nuclei are labeled with DAPI (blue). Scale bar: 200 μm.

**Figure 7:**
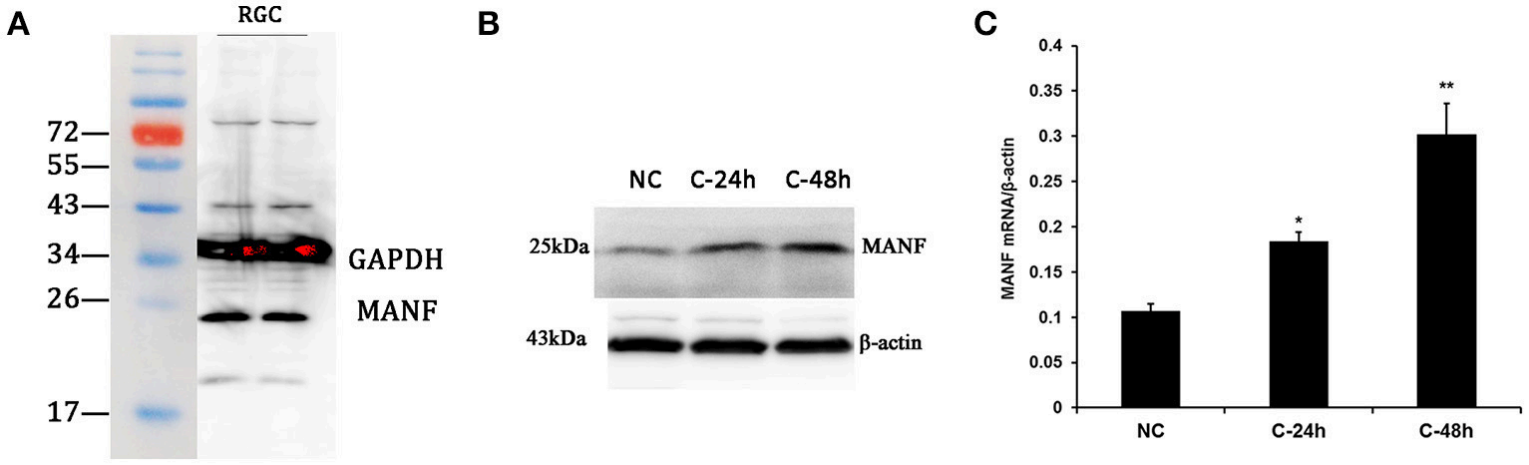
**Western blot analysis and real-time PCR analysis of MANF expression in cultured rat RGCs. (A)** Western blot, a major band of the expected molecular weight (25 kDa) is clearly visible. **(B)** Western blot analysis of MANF in RGCs incubated with 200 mM cobalt chloride (CoCl_2_) for 24 and 48 h. **(C)** Changes of MANF mRNA levels relative to the housekeeping gene β-actin (determined by real-time PCR) in RGCs incubated with 200 mM cobalt chloride (CoCl_2_) for 24 and 48 h (represented as the mean ± SEM, *n* = 6). ^*^P < 0.05, ^**^P < 0.01. NC: normal control; C-24 h/C-48 h: incubated with CoCl_2_ for 24/48 h.

Considering that primary culture of RGCs is a crucial and fundamental tool to study retinal physiological and pathophysiological mechanisms, and to further confirm the expression of MANF in RGCs, we purified and cultured RGCs for *in vitro* validation. The RGCs purity was about 85% after 3 days of culture (Figure 6) (Gao et al., 2016). To determine if RGCs express MANF, immunocytochemistry and western blot analyses were performed, and the results confirmed that MANF was robustly expressed in RGC (Figures 6, 7A). Further analysis of nuclear and cytoplasmic extracts revealed that MANF was detected primarily in the cytoplasmic extracts, with a substantial proportion in the nuclear fractions (Figure 5B, Brn 3a is a nuclear specific marker and Thy1 is a cytoplasmic specific marker Hu et al., 2010; Nadal-Nicolás et al., 2012). This is well consistent with the results of immunofluorescence labeling in retinal slices. We finally identified whether pathological conditions could affect the expression of MANF, RGCs were stimulated with 200 mM CoCl_2_ for 24 and 48 h. Western blot analysis and quantitative real-time PCR analysis both showed significant upregulation of MANF in the RGCs, wherein the mRNA levels were significantly elevated, 1.7-fold of the control level at 24 h and 2.8-fold at 48 h (*P* < 0.01, Figures 7B,C).

## Discussion

Numerous ophthamic diseases, such as diabetic retinopathy (Ikesugi et al., 2006; Li et al., 2009), glaucoma (Zode et al., 2015), retinal detachment (Liu et al., 2010; Zhu et al., 2013), and age-related macular degeneration (Li et al., 2008; Libby and Gould, 2010), are associated with neuronal apoptosis and ERS. Reducing ERS could be an underlying therapeutic strategy, where ERS manipulation could slow the progress of retinal degeneration and promote the survival of retinal neurons (Doh et al., 2010; Li et al., 2012, 2014). Studies have shown that MANF is a secretory protein and that ERS could induce its expression and secretion (Mizobuchi et al., 2007; Apostolou et al., 2008). It can be differently regulated by epileptic and ischemic insults in the rodent brain and heart (Apostolou et al., 2008; Lindholm et al., 2008; Tadimalla et al., 2008). Besides, large studies indicate that MANF can not only protect cultured nigral dopaminergic neurons and suppress cell proliferation and ERS-induced cell death, but also can affect cell morphology and size in non-neuronal cells (Petrova et al., 2004; Lindholm et al., 2007; Airavaara et al., 2009; Palgi et al., 2009; Voutilainen et al., 2009; Yu et al., 2010; Commissiong, 2012; Shen et al., 2012; Zhao et al., 2013; Yang et al., 2014; Cordero-Llana et al., 2015; Liu et al., 2015). Moreover, recently reported on science displayed that intravitreal injection recombinant MANF could promote alternative activation of innate immune cells, enhance neuroprotection and tissue repair, and improve the success of photoreceptor replacement therapies in the retina (Neves et al., 2016). These results implying that MANF may have a close relationship with the physical and pathological regulation of the retina. Further in-depth analysis of the expression and distribution of MANF in the retina and ON will provide more information and a basis for further studies about the effect of MANF in tissues and MANF could thus serve as a treatment modality for ophthalmic diseases in the immediate future.

In the present study, we observed that MANF could be expressed both in the retina and ON in rodents and humans, although with a smaller amount expressed in the rat ON. This is consistent with previous reports that MANF is highly expressed in neuronal tissues (Voutilainen et al., 2009; Chen et al., 2012). We did not carry out a detailed comparison of the expression of MANF in the human retina and ON owing to a lack of sufficient samples suitable for western blotting. However, we found that the level of MANF distribution in the retina is seemingly different between human and rodent. In rodents, MANF was distributed in cells within the GCL and INL and was mainly expressed in the GCL, preferentially in the cytoplasm of RGCs. However, in the human retina, MANF was mainly distributed in the GCL, with only mild staining seen in the INL and ONL. There are two possible explanations for this discrepancy in MANF expression between rodents and humans: either MANF expression is indeed mainly distributed in the GCL in human retinas, or the antibody used had greater affinity for rodent MANF than human MANF. In support of the second hypothesis, our real-time PCR and western blot data revealed that MANF mRNA and protein levels were lower in the human retina than in the rodent retina. With respect to the former hypothesis, MANF expression is more intense in the human retina than in the rodent retina, and hence, we are more inclined to accept the previous assumption. However, the results consistently show that MANF expression is highly conserved among mammals by the protein level seen in RGCs, suggesting that MANF may play a pivotal role in the functional regulation of RGCs in health and disease. To further identify the role of MANF in RGCs, we purified and cultured RGCs for *in vitro* validation. The results not only further confirmed the expression of MANF in RGCs but also showed that hypoxia could induce changes in MANF expression. These results conclusively indicate that MANF very likely plays a crucial role in hypoxia signaling in RGCs or in other retinal neuropathies. However, future work should investigate if manipulation of MANF could affect RGC viability in disease models as further studies are required to uncover the underlying mechanisms.

The highlight of this study is that the expression and distribution of MANF was verified by human retina and ON based on animal experiments, which is of great significance for advanced exploration of human diseases. However, as it is difficult to obtain whole human eyes for research, we only studied three normal eyes form Chinese donors of 38, 17, and 41 years old. Therefore, whether the distribution or expression of MANF would change with aging and various retinal abnormalities is still uncertain. Besides, the ON of the eyes we obtained were very short, therefore, protein and cDNA extracted from it was insufficient for western blotting or other analysis, and we only performed immunofluorescence staining. Further in-depth analysis is essential for better understanding.

To our best knowledge, this is the first report to verify the expression and distribution of MANF in the retina and ON in both human and rodent retinas. In combination with our recent study of MANF, these results are encouraging to undertake further studies regarding the regulation of ocular tissue in development, health, and disease and the MANF receptor and its signaling mechanisms so that MANF could serve as a potential treatment modality in the near future.

## Author contributions

JW and QW: Comprehensive arrangement for all aspects of the work and final approval of the version to be published; FG: Acquisition, analysis, and interpretation of data for the work, drafting the manuscript and revising it. TL and SZ: Acquisition the data.

### Conflict of interest statement

The authors declare that the research was conducted in the absence of any commercial or financial relationships that could be construed as a potential conflict of interest.

## Supplementary material

Supplementary Figure S1 **Changes in VEGF release (determined by ELISA) in RGC-5 cells treated with Cocl_2_ of different concentrations for 24 h**. ^**^*P* < 0.01 vs. the untreated control (Du et al., 2013). Changes in VEGF release (determined by ELISA) in RGC-5 cells treated with Cocl_2_ of different concentrations for 24 h. ^**^*P* < 0.01 vs. the untreated control (Du et al., 2013).

Supplementary Figure S2 **Negative controls**. Retinal and RGC samples incubated with secondary antibodies without primary antibody displayed little or no autofluorescence.
