# Supplementary figures and images for: Expression and Distribution of Mesencephalic Astrocyte-Derived Neurotrophic Factor in the Retina and Optic Nerve
Source: Front Hum Neurosci. 2017 Jan 19;10:686. doi: 10.3389/fnhum.2016.00686 (PMC5243802; doi:10.3389/fnhum.2016.00686)

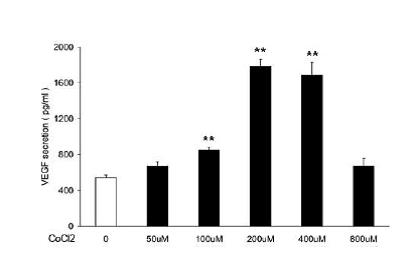

Supplement: Supplementary Figure S1 — Changes in VEGF release (determined by ELISA) in RGC-5 cells treated with Cocl2 of different concentrations for 24 h. **P < 0.01 vs. the untreated control (Du et al., 2013). Changes in VEGF release (determined by ELISA) in RGC-5 cells treated with Cocl2 of different concentrations for 24 h. **P < 0.01 vs. the untreated control (Du et al., 2013). [file Image1.jpg]

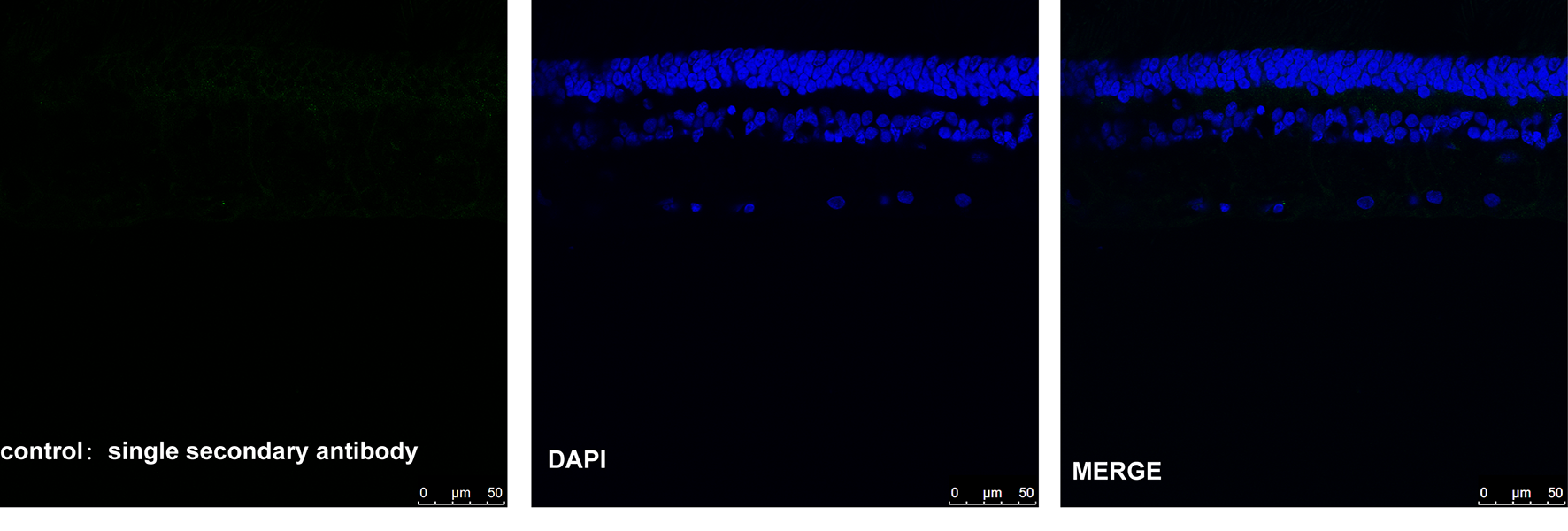

Supplement: Supplementary Figure S2 — Negative controls. Retinal and RGC samples incubated with secondary antibodies without primary antibody displayed little or no autofluorescence. [file Image2.tif]
